# Micronized natural progesterone (Seidigestan^®^) vs GnRH antagonists for preventing the LH surge during controlled ovarian stimulation (PRO_NAT study): study protocol of a randomized clinical trial

**DOI:** 10.3389/fendo.2024.1350154

**Published:** 2024-03-21

**Authors:** M. Martínez-Moya, J. Guerrero, J. L. Girela, A. Pitas, A. Bernabeu, R. Bernabeu, J. C. Castillo

**Affiliations:** ^1^ Bernabeu Institute, Alicante, Spain; ^2^ Biotechnology Department, Alicante University, Alicante, Spain

**Keywords:** progestin-primed ovarian stimulation (PPOS), GnRH antagonist protocol, IVF, oocyte donation, pregnancy

## Abstract

**Introduction:**

Progesterone-primed cycles effectively suppress the pituitary LH surge during ovarian stimulation in oocyte donors and in the infertile population. Particularly in oocyte donors, the use of synthetic progesterone (progestins) has been explored in prospective clinical trials, showing mixed results. This trial was designed to determine whether the use of micronized natural progesterone is as effective as the GnRH-antagonist protocol in terms of the number of mature oocytes (MII) retrieved in oocyte donation cycles as a primary outcome, and it also aims to explore the corresponding results in recipients as a secondary outcome.

**Methods:**

We propose a prospective, open-label, non-inferiority clinical trial to compare a novel approach for oocyte donors with a control group, which follows the standard ovarian stimulation protocol used in our institution. A total of 150 donors (75 in each group) will be recruited and randomized using a computer algorithm. After obtaining informed consent, participants will be randomly assigned to one of two ovarian stimulation protocols: either the standard GnRH antagonist or the oral micronized natural progesterone protocol. Both groups will receive recombinant gonadotropins tailored to their antral follicle count and prior donation experiences, if any. The primary outcome is the number of mature metaphase II (MII) oocytes. Secondary measures include treatment duration, pregnancy outcomes in recipients, as well as the economic cost per MII oocyte obtained in each treatment regimen. Analyses for the primary outcome will be conducted in both the intention-to-treat (ITT) and per-protocol (PP) populations. Each donor can participate only once during the recruitment period. The estimated duration of the study is six months for the primary outcome and 15 months for the secondary outcomes.

**Discussion:**

The outcomes of this trial have the potential to inform evidence-based adjustments in the management of ovarian stimulation protocols for oocyte donors.

**Clinical trial registration:**

ClinicalTrials.gov, identifier, NCT05954962.

## Highlights

• First randomized controlled study comparing the effectiveness of micronized natural progesterone (Seidigestan®) with the GnRH-antagonist protocol in terms of the number of mature oocytes (MII) retrieved in oocyte donation cycles. The primary outcome focuses on this comparison, with a secondary aim of exploring corresponding results in recipients.
• The trial is designed with several strengths, including a randomized and controlled structure to minimize bias. Furthermore, its double-center approach enhances the generalizability of the results. The use of fresh blastocyst-stage embryo transfers will provide an opportunity for a secondary examination of both embryo quality and the ongoing pregnancy rate in recipients.
• The outcomes of this trial carry the potential to significantly influence the field by providing evidence-based insights. Successful results could lead to adjustments in the management of ovarian stimulation protocols for oocyte donors.

## Introduction

The advent of controlled ovarian stimulation dates back to the 1980s when gonadotropins and clomiphene citrate were initially employed. However, this approach often led to the occurrence of endogenous LH surges in up to 50% of cases (1). Such LH surges triggered ovulation, resulting in a subsequent rise in serum progesterone levels, making it impossible to proceed with the necessary oocyte retrieval for *in vitro* fertilization.

To address this issue, GnRH agonists were introduced in the 1980s (2). These agonists, despite causing an initial and transient surge in both FSH and LH (known as the flare-up effect), eventually led to the suppression of pituitary GnRH receptor secretion (3). This breakthrough allowed for the recruitment of a greater number of follicles without the risk of premature luteinization, thereby improving the success rates of *in vitro* fertilization and enabling the scheduling of stimulation cycles (4). However, the use of GnRH agonists came with trade-offs, including higher exogenous gonadotropin expenses due to the lack of endogenous secretion, longer stimulation periods, and the requirement for the final oocyte maturation trigger using human chorionic gonadotropin.

The solution to these drawbacks came with the discovery of the third generation of GnRH antagonists, such as Cetrorelix and Ganirelix, which did not carry the risk of anaphylactic reactions seen in previous generations (5). These compounds allowed for the suppression of pituitary gonadotropin production in a reversible manner. For instance, discontinuing the administration of Ganirelix at a dose of 0.25 mg resulted in the complete disappearance of its effects within 24-27 hours (6). As a result, they enabled shorter stimulation periods (requiring lower doses of exogenous gonadotropins), reduced costs, and also facilitated the use of GnRH agonists with their flare-up effect for oocyte trigger, leading to a significant decrease in overall rates of ovarian hyperstimulation syndrome (7).

In 2015, the use of progestogens to suppress the LH surge in the follicular phase was first published, offering a cost-effective alternative to antagonists in ovarian stimulation (8). This approach also simplified the process by transitioning from subcutaneously administered antagonists to orally administered medications. Subsequently, three randomized clinical trials have been published, demonstrating the effectiveness of continuous use of dydrogesterone (9) and medroxyprogesterone acetate or MPA (10, 11) as progestogens capable of inhibiting the LH surge.

To date, there has been no randomized study comparing the use of micronized natural progesterone (MNP) to the current standard treatment, namely, the antagonist protocol. We have retrospective data suggesting its similarity to treatment with MPA (12) in terms of the number of oocytes retrieved, as well as prospective data indicating its non-inferiority compared to dydrogesterone (13). This study represents the first randomized trial comparing Seidigestan® as MNP to the antagonist protocol.

## Outcomes

The primary objective of this study is to compare the efficacy (non-inferiority) of the MNP protocol (Seidigestan®) to an antagonist protocol, using the number of mature metaphase II (MII) oocytes as the primary outcome variable.

Our secondary objectives are detailed in **Table 1**.

**Table 1:** Secondary outcomes for comparison of oral progesterone vs GnRH antagonist.

Treatment	Analyzing and comparing the baseline characteristics of each group of donors
	Comparing the gonadotrophin dose and treatment duration
Laboratory	Fertilization rate per-oocyte injected
	Assessing whether there are differences in the performance of these MII oocytes in fresh cycles and their potential to develop into blastocysts after ICSI
Clinical	Documenting the fresh transfers of blastocysts originating from each protocol and the endometrial preparation protocols
	Biochemical pregnancy: a pregnancy diagnosed only by the detection of beta hCG in serum or urine, which fails to progress to the point of ultrasound confirmation
	Clinical pregnancy: an intrauterine pregnancy diagnosed by ultrasonography
	Ongoing pregnancy: the presence of a heartbeat as seen by ultrasonography 12 weeks gestation
	Multiple pregnancy: two or more gestational sacs seen by ultrasonography
	Early pregnancy loss: the spontaneous loss of an intrauterine pregnancy, where there is no fetal heartbeat detected at the time of ultrasound at 6–8 weeks
	Ectopic pregnancy: a pregnancy outside the uterine cavity, diagnosed by ultrasound, surgical visualization or histopathology
	Miscarriage: the spontaneous loss of an intrauterine pregnancy with fetal heartbeat prior to 20 completed weeks of gestational age
	Live birth: the complete expulsion or extraction from a woman of a product of fertilization, after 22 completed weeks of gestational age; which, after such separation, breathes or shows any other evidence of life, such as heartbeat, umbilical cord pulsation or definite movement of voluntary muscles, irrespective of whether the umbilical cord has been cut or the placenta is attached
	Analyzing possible obstetric differences between both protocols
Cost-effectiveness	Contrasting the economic cost per number of mature oocytes (MII) obtained in each treatment regimen
Quality of life	Women will also be asked to complete a survey consisting of questions related to treatment tolerability

## Materials and methods

### Study design

**Figure 1:**
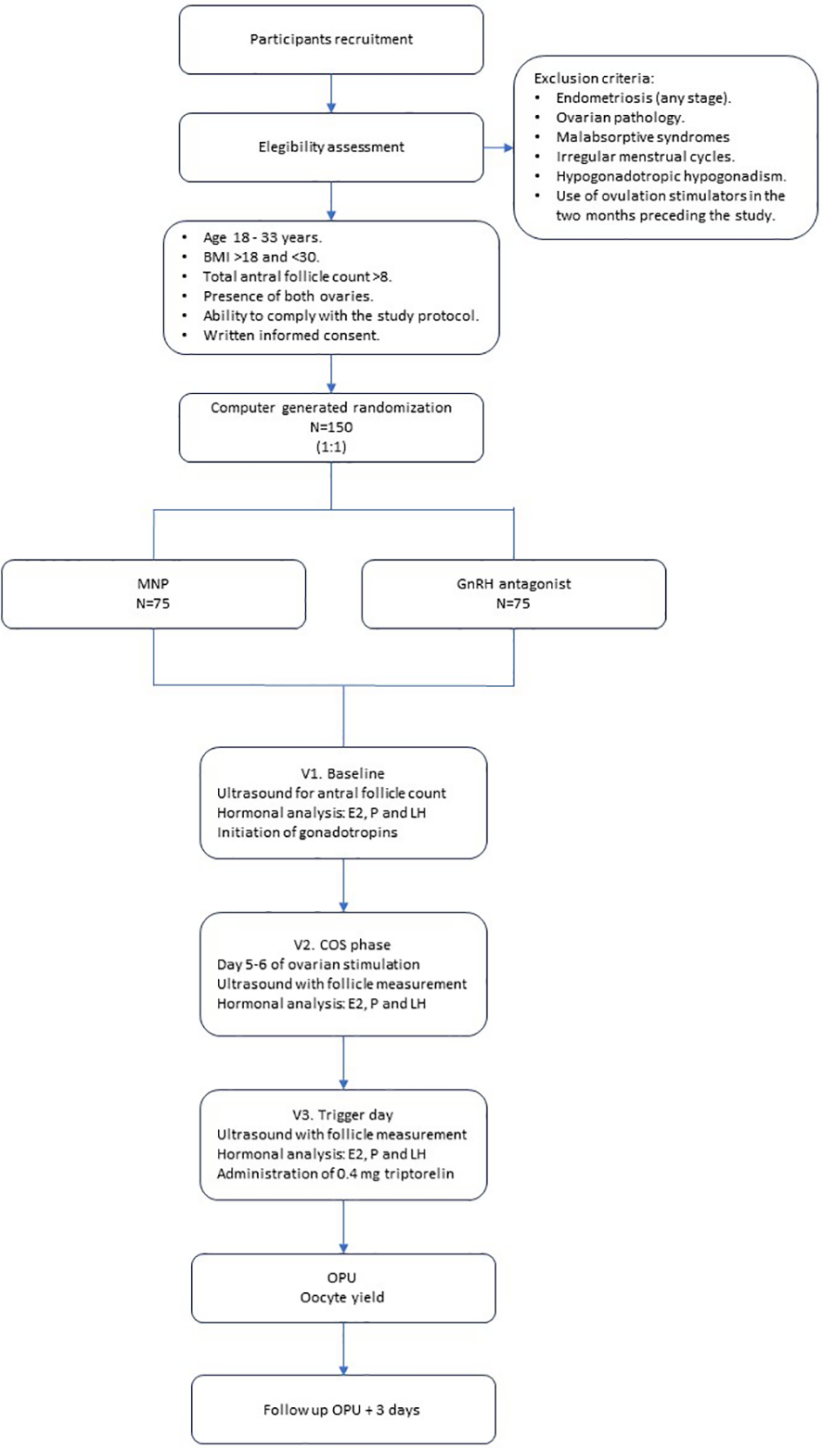
Study flow diagram.

To address both the overarching and specific objectives, we have designed a prospective, randomized, non-inferiority comparative clinical trial, conducted in an open-label fashion, with a control group following the standard therapeutic protocol at our institution for ovarian stimulation in oocyte donors during the year 2023. This multicenter study involves the locations in Alicante and Elche, which are part of IB S.L. (Instituto Bernabeu S. L), as well as the gynecology center Accuna (Namsomara S.L). The observer (the gynecologist conducting the ultrasound) will be blinded to the patient’s group allocation, and the person responsible for data analysis will also be blinded to group assignment. The blinding may be removed if the physician responsible for the subject deems it necessary for safety reasons. **Figure 1**.

#### Oocyte donors and ovarian stimulation

After concluding the suitability assessment processes, potential oocyte donors will be approached for their willingness to participate in the study. They will be provided with an explanation of the study and presented with the informed consent form and patient information sheet. Random allocation has been executed using the SPSS Statistics macro!RNDSEQ (Domenech JM. Generation of Random Sequences [computer program]. V2011.09.09. Bellaterra: Universitat Autònoma de Barcelona; 2011), ensuring that both study groups have an equal likelihood of assignment at each instance. The researchers will not have access to the randomization list. The designated individual responsible for randomization will open each patient’s randomization envelope (which is opaque) and allocate the corresponding medication (Seidigestan® oral vs. Ganirelix subcutaneous). Patients will not be blinded to their assigned group due to the differing routes of medication administration, and neither will the randomization administrator nor the nursing staff. Participants in this study will not receive any specific additional financial compensation.

The patient will notify the clinic on the first day of her menstrual cycle and will be scheduled for an appointment during the initial four days of her cycle. During this visit, an ultrasound examination will be conducted to ensure there are no contraindications to commence ovarian stimulation. If no contraindications are found, the administration of the prescribed dose of gonadotropin (Bemfola®) will commence, and this day will be considered the first day of stimulation. The gonadotropin starting dose was selected to balance follicular recruitment optimization and minimize the risk of high response. To summarize, the suggested optimal dose was 150 IU for donors with an antral follicle count (AFC) greater than 14, while a dose of 225 IU was deemed suitable for donors with 10-14 antral follicles. In cases where fewer than 10 follicles were observed, a dose of 300 IU was determined. It’s important to note that, in line with clinician discretion, these doses could be adjusted based on the donor’s BMI, and responses to previous stimulations (when appropriate).

If the donor has been on oral contraceptive pills (OCPs) in the previous month, she will be scheduled for a baseline ultrasound examination (Visit 1) between 5-7 days after discontinuing the OCPs. Ovarian stimulation will commence on this day.

*For the study group*, starting on this day, they will begin taking one 200 mg soft capsule of Seidigestan® in the evening, a regimen that will continue until the day of the GnRH agonist (Decapeptyl® 0.4 mg). At this point, a blood sample will be collected for subsequent analysis of baseline hormone levels (LH, estradiol, and progesterone) **Table 2**.

**Table 2:** Overview of study visits.

	Enrollment / Baseline	COS phase	GnRH agonist trigger	OPU	Follow-up OPU + 3 days
Information and counselling	X				
Signing of informed consent	X				
Treatment related data collection	X	X	X	X	X
Randomization	X				
Transvaginal ultrasound scan	X	X	X		
Blood sample	X	X	X		

*Both groups* will be scheduled for a follow-up ultrasound and blood analysis on day 5-6 of stimulation. *For participants in the control group*, the nurse will instruct them on the initiation of the GnRH antagonist (Ganirelix. Astarté® 0.25 mg) according to a fixed day 5/6 protocol. This regimen will be maintained until the day of administration of the GnRH agonist (Decapeptyl® 0.4 mg) to trigger final oocyte maturation. During ovarian stimulation controls, doctors in charge of subjects have freedom to change gonadotropin dosage if needed.

In the event that more than 26 hours have elapsed since the last Ganirelix injection until the time of agonist administration, the patient will be advised to administer an additional dose of Ganirelix due to the risk of falling below the minimum effective blood concentration (6). This additional dose should be administered at least 7 hours before the scheduled time for the analog administration (14).

Following this, participants will be scheduled for follow-up ultrasound and blood analyses every 24-72 hours as determined by the medical team.

Ovarian stimulation will be deemed complete when more than 6 follicles of equal or greater than 18 mm in size are observed. On the same day, the final oocyte maturation will be triggered by administering 0.4 mg of triptorelin acetate (Decapeptyl® 4 ampoules), the final study blood sample will be obtained, and the oocyte retrieval will be scheduled 36 hours later, following the center’s standard protocol.

At each visit, participants will be asked about the occurrence of any adverse effects or new symptoms, as well as any concurrent medication they may be taking. Moreover, participants will return used injectables and given blisters of oral progesterone in each consultation to monitor compliance with the protocol.

Ovarian stimulation will be discontinued in the event of poor response (*i.e.* ≤ 8 follicles ≥ 14 mm), and consequently, the administration of any medication will cease if the criteria for final oocyte maturation are not met within a maximum of 14 days from the initial gonadotropin dose administration.

The final visit will involve a telephone call conducted 72 hours after the oocyte retrieval to gather information regarding any surgical or stimulation-related complications.

Our donor program ensures a minimum of 8 metaphase II oocytes per recipient. Therefore, cumulus-corona-oocyte complexes (CCOs) are decumulated on the day of the donor’s oocyte retrieval and fertilized using intracytoplasmic sperm injection (ICSI). Only in exceptional cases, as determined by medical judgment, where conventional IVF may be more beneficial, will the decumulation process not be performed. All embryo transfers will occur at the blastocyst stage.

### Blood sample collection during the trial

Blood samples will be drawn during trial visits that include vaginal ultrasounds. These samples will be analyzed for estradiol, progesterone, and LH levels.

#### Recipients and endometrial preparation

Recipients will be between 18 and 50 years old at the time of embryo generation with different indications to receive donor oocytes: advanced maternal age, failed IVF cycles with own oocytes, failed insemination cycles, low ovarian reserve, endometriosis, recurrent miscarriage, genetic or chromosomal abnormalities transmissible to offspring and spontaneous or iatrogenic menopause, with a BMI of <30 kg/m² and without systemic illnesses that could be exacerbated during pregnancy or pose a risk to the patient in the event of gestation. In cases where there are uncertainties, as determined by the attending physician, or in recipients older than 45 years, a report from their primary care physician or responsible specialist certifying their good health for pregnancy will be required. To comprehensively study the clinical outcomes, we plan to retrospectively gather and analyze baseline information on recipients. The collected medical and gynecological data will include the following parameters: Age, BMI, uterine pathologies (endometriosis and adenomyosis, specifying the severity of these conditions), fibroids (type and measurement), immunological or hematological diseases, obstetrical history, endometrial thickness during the endometrial preparation phase and serum progesterone level prior to the transfer procedure.

For recipients with ovarian function, the hypophysis will be suppressed with GnRH agonists (Triptoreline, 3.75 mg, Decapeptyl 3.75 mg, Ipsen Pharma). This step will be omitted in patients with inactive ovaries. Subsequently, hormonal replacement therapy with transdermal estradiol or oral estradiol valerate will be initiated, following a step-up regimen, for a minimum of 10 days. An additional transvaginal ultrasound will be performed to assess endometrial thickness and pattern. If the physician deems it optimal, progesterone supplementation will be introduced (while continuing the estrogen regimen) either as 800 mg daily vaginal progesterone, as described previously by our group (15). Before the transfer, progesterone levels will be measured between days 3-5 of progesterone administration to customize the dosage for each patient.

In cases where a male partner is involved, a seminogram is conducted to evaluate basic sperm parameters. Based on the findings, the screening may be expanded to include additional tests for a more thorough analysis: Karyotype, DNA fragmentation test, testicular sonography (in instances where pathology is observed or suspected), Sperm Fluorescence *In Situ* Hybridization (FISH). If obstructive azoospermia is identified, a cystic fibrosis screening is conducted to assess the risk associated with this condition. Finally, we offer a genetic compatibility test, aiming to reduce the risk of recessive genetic diseases in the offspring.

### Study population

The study population comprises women undergoing controlled ovarian stimulation at Centers Accuna e Instituto Bernabeu Elche. Our study sample will be selected based on the following criteria:

Inclusion Criteria: Patients included in the study must meet the following inclusion criteria:

O Eligibility for participation in the Instituto Bernabeu’s oocyte donation program.
O Age between 18 and 33 years.
O BMI >18 and <30.
O Antral follicle count >8 (combined count from both ovaries).
O Presence of both ovaries.
O Ability to participate and comply with the study protocol.
O Proficiency in spoken and written Spanish.
O Provided written informed consent.

Exclusion criteria: Patients meeting any of the following criteria will not be included in the study:

O Diagnosis of endometriosis (any stage).
O Diagnosis of any ovarian tumor, whether benign or malignant.
O Concurrent participation in another study.
O Malabsorptive syndromes that may affect the efficacy of Seidigestan®, such as bariatric surgery, ulcerative colitis, or Crohn’s disease.
O Patients with irregular menstrual cycles.
O Patients with hypogonadotropic hypogonadism.
O Received treatment with ovulation stimulators in the two months preceding the study.
O Previously participated in the current study.

### Statistical analyses

#### Sample size

Considering an expected yield of 13.58 oocytes in the control group with a standard deviation of 6.997 (based on an internal study conducted within the clinic, encompassing all oocyte donor stimulations performed in 2021), and accepting an alpha risk of 0.05 and a beta risk of 0.20 in a one-sided test (with an 80% statistical power), a sample size of 136 patients (68 in each study group) is required to detect a minimum difference of 3 oocytes. Accounting for an estimated loss of 10%, a total sample size of 150 patients (75 per group) is needed.

#### Data analysis

The descriptive statistical methods employed in this study will depend on the nature of the analyzed variables. For qualitative variables, the following descriptive statistics will be computed: frequency and percentage. Quantitative variables will be presented using measures of central tendency and dispersion.

For univariate statistical analysis of qualitative variables, the Chi-square test or Fisher’s exact test will be utilized. Normality of quantitative variables will be assessed through the Kolmogorov-Smirnov and Shapiro-Wilk tests, as well as visual examination of distribution (histogram, QQ-plot, and box plot). If the distribution is found to deviate from normality, logarithmic transformation of the variable will be applied. Subsequently, univariate analysis of quantitative variables will be performed using the t-Student test. Statistical significance will be considered at P < 0.05, and conclusions will be drawn with support from confidence intervals whenever applicable.

The primary objective of the study will be analyzed through an ANCOVA, using the treatment group assignment as a covariate, to determine whether there is an equivalent effect among subgroups or not.

For the analysis of secondary objectives, a multilevel model will be employed, considering the hierarchical structure of the data due to the fact that one donor may contribute to multiple recipients, as well as the number of cycles.

Data will be entered into a database within our department and analyzed using statistical software packages SPSS version 22.0 for Windows (SPSS Inc., Chicago, IL) and R version 4.2.2 for Windows (R Foundation for Statistical Computing, Vienna, Austria).

#### Analysis of the primary outcome

The analysis of the primary variable will be presented as the mean number of oocytes along with its corresponding 95% confidence interval. Student’s t-test will be used to compare the mean number of oocytes between each group. A multivariate linear model will be adjusted for the number of oocytes, incorporating the group and potentially confounding variables. A variable selection process using stepwise selection based on the AIC (Akaike Information Criterion) will be conducted.

The primary analysis will be performed in the intention-to-treat (ITT) analysis population and will also be repeated in the per-protocol (PP) analysis population.

#### Analysis of secondary outcomes

The secondary objectives will be analyzed based on the nature of the variable under evaluation. Parametric tests, such as Student’s t-test, or non-parametric tests, such as the Wilcoxon-Mann-Whitney test for independent data, will be employed for secondary analyses comparing the two treatments, depending on the normality or non-normality of the quantitative data distribution. For categorical variables, the Chi-square test or Fisher’s exact test, as appropriate, will be used.

Secondary analyses will be conducted in the intention-to-treat (ITT) analysis population.

Given that randomization has been carried out with oocyte donors rather than recipients, caution must be exercised when interpreting the results for the latter. To mitigate potential biases and control for the possibility of a single donor contributing oocytes to multiple recipients, the impact of different treatments will be assessed using a multilevel analysis. This analysis will consider the hierarchical or nested structure of our data and will be multivariable, incorporating various covariates to control for potential biases.

### Data management and monitoring

The data collected must be transcribed into the Data Collection Notebook (DCN), and this data will be considered as valid information for the subsequent evaluation of the efficacy and safety of the treatments under study. The DCNs should be completed accurately, and in cases where previously transcribed data need correction, they should be crossed out with the correct value noted beside them. Corrections must always be dated and validated by the signature of the principal investigator or their collaborators.

The documents pertaining to this trial will be archived by the investigator for up to five years following the completion of the clinical trial. In any case, a participant identification list will always be maintained. The sponsor, Instituto Bernabeu, will keep the primary study archive for a period of five years.

Monitoring of the study is expected to be conducted in accordance with the procedures outlined in the Standard Operating Procedure (SOP) of the monitoring and follow-up Unit of the institute.

The controlled ovarian hyperstimulation cycles of the oocyte donors will be monitored according to the center’s standard criteria.

Regarding the handling of personal data in the study, such data will be processed and safeguarded by qualified members of the medical team with the utmost confidentiality, in compliance with the prevailing data protection regulations, including Regulation (EU) 2016/679 of the European Parliament and of the Council of 27 April 2016 on the protection of natural persons with regard to the processing of personal data and on the free movement of such data (GDPR), and the Spanish Organic Law 3/2018 of 5 December on the Protection of Personal Data and the Guarantee of Digital Rights (LOPDGDD), as well as any other relevant regulations and legislation.

To safeguard the confidentiality of the subjects’ personal data, only the principal investigator, their collaborators, and the technical personnel involved in the study will have access to their identities but they will not be disclosed to sponsors. For the same reason, complete personal identification data and written consent will be stored in the investigator’s center archive. Information regarding the identity of the patients will be considered confidential for all purposes. Patient data collected in the CRD during the study will be documented in an anonymous and dissociated manner, linked to a code (patient number), so that only the investigator can associate such data with an identified or identifiable person. The database generated by the study will not contain any patient identification, only a numerical code that cannot be used to reveal their identity. The information collected in the study will always be treated as aggregated data and never as individual or personal data, thus maintaining anonymity and confidentiality.

### Ethical approval

The project has been approved by the Ethics Committee of the University General Hospital of Elche in May 2023 (approval n°4/2023). This committee has certified that the ethical and scientific aspects related to the research project have been analyzed, confirming that it meets the appropriate characteristics regarding patient information and compliance with ethical criteria for medical and biomedical research established in the Helsinki Declaration (June 1964, Helsinki, Finland, modified in 2008) for conducting research projects.

Any subsequent amendments to the protocol should be reported to the Committee for Ethics in Drug Research (CEIm), and their opinion should be sought if a new assessment of the ethical aspects of the trial is required.

All patients will receive both informed consent in advance and an information sheet about the study’s conduct and potential risks. Participation will be entirely voluntary, and refusal will not have any repercussions for the donor.

### Date of first patient’s enrolment

Enrollment is planned for November 2023.

## Discussion

Since the mid-20th century, thanks to the production of synthetic progesterone from cholesterol, the ability of this molecule to inhibit ovulation has been known (16). Subsequent studies in mammals allowed understanding the interaction and function of progesterone with the hypothalamic-pituitary axis (17). It was demonstrated that continuous administration of progesterone inhibits the peak of gonadotropins due to its effect on the central nervous system (CNS) and not in the pituitary as previously believed (18). This fact has been utilized in the creation of contraceptives containing only progestin derivatives and, due to its impact on the endometrium, in therapeutic schemes for endometrial pathology in women who wish to preserve their fertility or whose baseline condition is not favorable for surgical intervention (19).

Pharmacologically, there are various progestins, whether containing natural or synthetic progesterone or one of its derivatives. Initially, orally administered natural progesterone was not useful due to its high hepatic metabolism (over 80%) and poor absorption. However, by reducing particle size through micronization and presenting it in a gelatin capsule with an oily vehicle, its blood levels significantly increase. This MNP reaches its peak concentration about 4 hours after ingestion, lasts up to 24 hours in the blood, and its absorption is enhanced by food intake (20).

Although there were previous studies using progestins such as MPA (21) for the prevention of LH surge during controlled ovarian stimulation in 2014, the first time MNP was used orally for this purpose was in 2015 when Zhu X (22) published a comparative retrospective study where 187 women used this progesterone at a daily dose of 200 mg (two doses of 100 mg spaced 12 hours apart). They found no differences in the number of retrieved oocytes compared to the short agonist protocol. It is worth noting the increase in gonadotropin doses used in the progesterone protocol due to the extended number of days required before the trigger (8.3 vs 8.94, p>0.001). Later, the same group published a comparative randomized study between MNP doses of 100 mg vs 200 mg. While they found no differences in gonadotropin doses or incidence of LH peaks, there was a higher rate of mature oocytes in the 200 mg dose group (23).

From here, the use of oral progesterone or its derivatives has been spreading as a therapeutic tool in assisted reproductive techniques due to its convenience for patients, although not without drawbacks. Introducing progesterone acts, among other things, on the endometrium, preventing the fresh transfer of embryos and necessitating a freeze-all strategy. Therefore, its use is reserved for cases where embryos will be vitrified either due to preimplantation aneuploidy screening or anticipated high response, or in cases where oocytes will be vitrified for accumulation or for fertility preservation for future medical or social reasons.

A special case is women participating in oocyte donation programs, where it is crucial to ensure the most patient-friendly protocol for their comfort. In this case, endometrial decidualization is not a factor to consider since the eggs will be donated to recipient patients. In this context, Beguería et al. (10) conducted a clinical trial where they used 10 mg of MPA daily compared with the antagonist protocol in 252 oocyte donors. They confirmed that the number of mature oocytes was comparable without increasing either the days or the dose of gonadotropins. However, they found that while the results were comparable in terms of fertilization rates and embryos obtained, they differed in terms of obstetric outcomes (ongoing pregnancy 26.7% vs 39.9%, p=0.015) in favor of not using MPA. It’s worth noting that pregnancy rates were not their primary outcome, so the study was not designed for this, and the results contradict previous studies (24).

In a randomized clinical trial involving 100 patients with infertility, a comparison was made between the use of MNP (27) at a dosage of 100 mg every 12 hours and an antagonist protocol. Surprisingly, the results revealed a higher number of mature oocytes in cycles with progesterone (10.82 vs 6.96, p< 0.001). This finding contradicts existing medical literature, as three previously published randomized clinical trials demonstrated no significant difference in the mean number of retrieved oocytes when utilizing dydrogesterone (9) and MPA (10, 11) as progestogens to inhibit the LH surge. Additionally, it is important to note that this study, in our opinion, has methodological deficiencies. These include unclear descriptions of the randomization process and the reasons for cancellation in many cases, the non-inclusion of potential confounding variables in the analysis (such as the difference in the average age between groups), and imprecise descriptions of the day (cleavage vs blastocyst) or number of embryos transferred. Therefore, in our view, a study with superior methodology is still needed to provide solid evidence on the use of MNP as an agent to prevent premature LH surge in controlled ovarian stimulation cycles.

For all these reasons, we have decided to conduct the first clinical trial comparing a daily dose of 200 mg (in a single capsule) with the antagonist protocol in oocyte donors. The strengths of this trial include its randomized, controlled design, which should minimize bias, and a double-center design, which enhances the generalizability of the results. The obtained oocytes will be used fresh for blastocyst-stage embryo transfer, allowing us to explore, although as a secondary objective, the embryo quality and the ongoing pregnancy rate of both groups. The results may lead to evidence-based changes in the management of ovarian stimulation protocols in oocyte donors.

## Ethics statement

The studies involving humans were approved by Hospital General Universitario de Elche. The studies were conducted in accordance with the local legislation and institutional requirements. The participants provided their written informed consent to participate in this study. Written informed consent was obtained from the individual(s) for the publication of any potentially identifiable images or data included in this article.

## Author contributions

MM: Conceptualization, Investigation, Methodology, Resources, Supervision, Visualization, Writing – original draft, Writing – review & editing. JGu: Data curation, Formal analysis, Supervision, Writing – review & editing. JGi: Supervision, Writing – review & editing. AP: Funding acquisition, Project administration, Resources, Supervision, Writing – original draft. AB: Funding acquisition, Resources, Supervision, Validation, Writing – review & editing. RB: Funding acquisition, Resources, Supervision, Validation, Writing – review & editing. JC: Conceptualization, Funding acquisition, Writing – original draft, Writing – review & editing.
